# Correction to “Atrophic Scars, Maturation Time and Therapeutic Outcomes Following Microneedling: A Post Hoc Analysis of a Randomized Clinical Trial”

**DOI:** 10.1111/jocd.71214

**Published:** 2026-09-30

L. G. de Souza , M. R. Silva , D. Steiner , and G. Viana de Oliveira , “Atrophic Scars, Maturation Time and Therapeutic Outcomes Following Microneedling: A Post Hoc Analysis of a Randomized Clinical Trial,” Journal of Cosmetic Dermatology 25, no. 6 (2026). 10.1111/jocd.70943

The authors have notified us of an error in the second author's name. The name “Márcio Rodrigues da Silva” should be corrected to “Marcio Roberto Silva.”

The online version of this article has been corrected accordingly.

We apologize for this error.

